# PPP6C, a serine-threonine phosphatase, regulates melanocyte differentiation and contributes to melanoma tumorigenesis through modulation of MITF activity

**DOI:** 10.1038/s41598-022-08936-0

**Published:** 2022-04-02

**Authors:** Carolyn R. Maskin, Renuka Raman, Yariv Houvras

**Affiliations:** 1 Department of Surgery, Weill Cornell Medical College, New York Presbyterian Hospital, New York, NY USA; 2 Department of Medicine, Weill Cornell Medical College, New York Presbyterian Hospital, New York, NY USA

**Keywords:** Cancer, Cancer genetics, Skin cancer, Cancer genetics, Development, Gene expression, Gene regulation

## Abstract

It is critical to understand the molecular mechanisms governing the regulation of MITF, a lineage specific transcription factor in melanocytes and an oncogene in melanoma. We identified PPP6C, a serine/threonine phosphatase, as a key regulator of MITF in melanoma. PPP6C is the only recurrently mutated serine/threonine phosphatase across all human cancers identified in sequencing studies and the recurrent R264C mutation occurs exclusively in melanoma. Using a zebrafish developmental model system, we demonstrate that PPP6C expression disrupts melanocyte differentiation. Melanocyte disruption was rescued by engineering phosphomimetic mutations at serine residues on MITF. We developed an in vivo MITF promoter assay in zebrafish and studied the effects of PPP6C(R264C) on regulating MITF promoter activity. Expression of PPP6C(R264C) cooperated with oncogenic NRAS(Q61K) to accelerate melanoma initiation in zebrafish, consistent with a gain of function alteration. Using a human melanoma cell line, we examined the requirement for PPP6C in proliferation and MITF expression. We show that genetic inactivation of PPP6C increases MITF and target gene expression, decreases sensitivity to BRAF inhibition, and increases phosphorylated MITF in a BRAF(V600E) mutant melanoma cell line. Our data suggests that PPP6C may be a relevant drug target in melanoma and proposes a mechanism for its action.

## Introduction

The incidence of melanoma has tripled worldwide over the past 30 years and remains one of the top five leading causes of new cancer cases and deaths^1^. While early disease stages are treatable with surgery, metastatic melanoma remains a deadly disease^2^. Recently, significant advances in targeted and immune therapies have been made. Targeted therapy using small molecule inhibitors to target BRAF and MEK have improved patient survival^3,4^. Unfortunately, half of all patients do not respond to currently available therapies and ultimately succumb from metastatic disease, underscoring the need to identify new drug targets.

Next-generation sequencing has brought unprecedented insight into the genomic alterations in melanoma. One recurrent mutation identified occurred in the catalytic subunit of a protein serine-threonine phosphatase complex, *PPP6C*^5,6^*.* Protein phosphatase 6C (PPPC6) is a member of the serine/threonine protein phosphatase family, which is conserved throughout eukaryotes. The PP6 complex is widely expressed in many tissue types and has been implicated in diverse processes such as cell cycle, inflammatory signaling, and lymphocyte development^7–9^. Recent studies suggest that PPP6C mutations affect its activity and participate in melanoma tumorigenesis through resulting MEK hyperphosphorylation^10^. However, its role in a melanocyte-specific developmental context has not been studied extensively, and the role that the R264C recurrent mutation plays in disease progression has not been the focus of intense study.

Melanocytes are reliant on regulating MITF expression, a critical transcription factor^11^. MITF is highly expressed during development and upregulates a transcriptional program promoting melanocyte differentiation^12^. Regulation of the activity and expression level of MITF is in part controlled by its phosphorylation^13^. KIT-mediated ERK signaling can phosphorylate MITF, leading to an increase in its expression^14^. The phosphatases that target MITF for dephosphorylation remain unknown.

In melanoma, MITF can function as a lineage survival oncogene, where unregulated expression can transform melanocytes^15^. However; MITF expression varies across melanomas, producing different melanoma phenotypes^16^. At lower levels of expression, MITF upregulates other transcriptional programs such as increasing expression of DIAPH1 leading to suppression of CDKN1B^17^. In melanomas, an MITF-low protein level is associated with invasion and proliferation^18^ and has been shown to be relatively resistant to immunotherapies^19^. Therefore, modulation of MITF during melanoma tumorigenesis and progression remains an important area of study.

In this study, we have defined a novel function for *PPP6C* during melanocyte development and implicated *PPP6C*(R264C) in accelerated melanoma tumorigenesis. Using genetic approaches, we have explored the consequences of gain and loss of PPP6C function on melanocyte specification, proliferation, and the development of melanoma in vivo. These data suggest that the phosphatase PPP6C blocks melanocyte differentiation and the recurrent R264C mutation is a gain of function alteration that leads to enhanced tumorigenesis.

## Results

### PPP6C is recurrently mutated in melanoma and has a unique relationship with the melanocyte lineage and MITF

**Figure 1:**
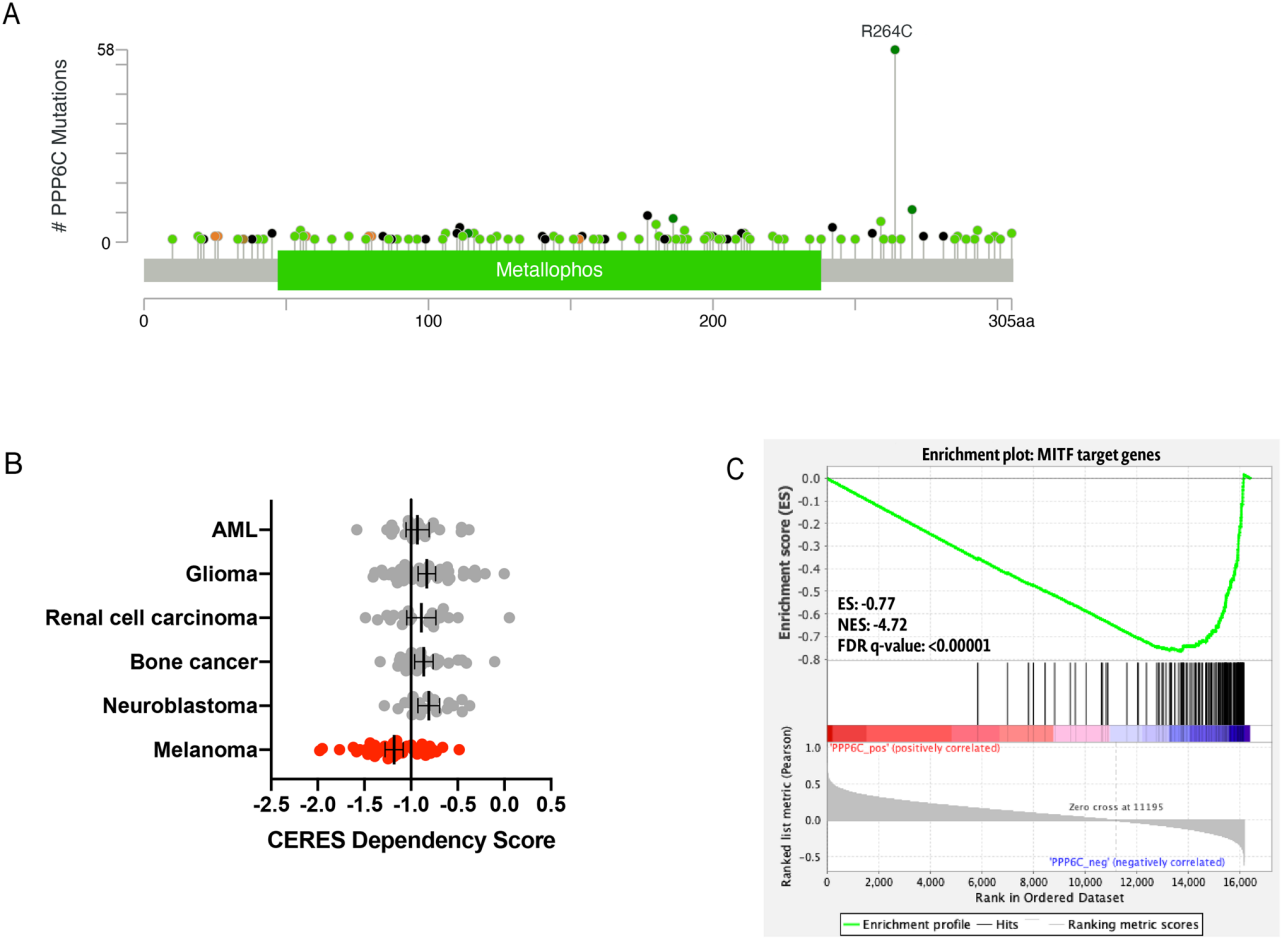
PPP6C is recurrently mutated in melanoma and has a unique relationship to melanocyte lineage and MITF. (**A**) Lollipop plot showing the distribution of PPP6C mutations across the coding protein. The y-axis represents the number of mutations. Data represents PPP6C mutations found in 184 pan-cancer studies. 57/58 tumors containing a PPP6C(R264C) mutation were melanomas. (**B**) Dependency scores derived from CRISPR loss of function screens across multiple cancer and primary cell lines. A gene with a score of less than − 1 is considered essential in that cellular context. (**C**) Gene set enrichment analysis of the transcriptomes of 42 publicly available melanoma cell lines. Plot represents MITF target gene enrichment across increasing PPP6C expression.

The Cancer Genome Atlas Project performed next generation sequencing on 331 primary and metastatic melanomas. Mutations in *PPP6C* were identified in 7% (n = 24) of tumors sequenced. Of those, 36% (n = 9) harbored a R264C mutation. PPP6C(R264C) co-occurred with both *BRAF*(V600E) and *NRAS*(Q61K) mutations^5^. We further analyzed PPP6C mutation data from 48,000 tumors across 184 pan-cancer studies. This analysis reveals that PPP6C mutations were identified in 285 tumors. Overall, 22% (n = 58) of PPP6C-mutated tumors contained the hot-spot *PPP6C*(R264C) mutation. All but one of these tumors containing a PPP6C(R264C) mutation were melanomas, suggesting a unique requirement for *PPP6C* in melanocytes (Fig. 1A). PPP6C appears to be the only recurrently mutated serine threonine phosphatase across all human cancers^20^, suggesting it plays an important role in melanoma regulation.

We leveraged publicly available CRISPR loss of function screens across a diverse array of human primary and immortalized cell lines to investigate the requirements for PPP6C in proliferation^21^. Melanoma cell lines are uniquely dependent on PPP6C expression for proliferation (Fig. 1B). Based on this data, we suspected a unique relationship between PPP6C and the melanocyte lineage. MITF is the critical transcriptional regulator of melanocyte development and upregulates a set of genes to drive differentiation^11,12^. In order to explore whether there was a link between the transcriptional program of MITF and PPP6C, we investigated 42 publicly available melanoma cell lines from the Broad Institute^22^. Our analysis reveals that PPP6C expression negatively correlates with MITF target gene expression (Fig. 1C). These findings suggest that PPP6C may play a previously unexamined role in MITF regulation in the melanocyte lineage.

### PPP6C disrupts melanocyte differentiation in vivo

**Figure 2:**
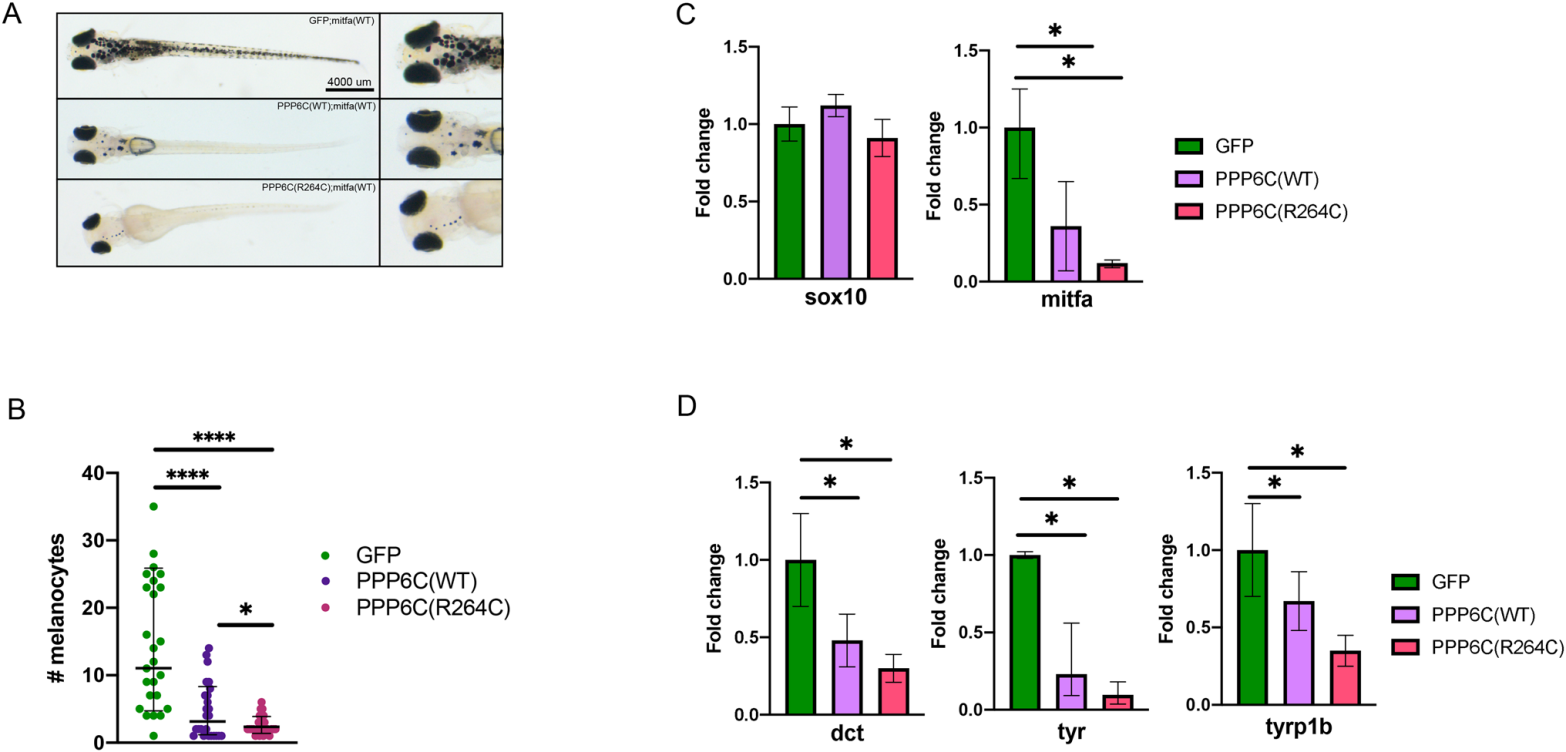
PPP6C disrupts melanocyte differentiation in vivo*.* (**A**) Representative images of 5 day old fish on the roy;nacre background injected with either MC-GFP, MC-PPP6C, or MC-PPP6C(R264C). (**B**) Representative counts of melanocytes of 5 day old fish on the roy;nacre background injected with either MC-GFP, MC-PPP6C, or MC-PPP6C(R264C) (Student’s two-tailed t-test). (**C**) Real-time qPCR measurement of SOX10 and MITF in 48 h post fertilization fish injected with either MC-GFP, MC-PPP6C or MC-PPP6C(R264C) (Student’s two-tailed t-test). (**D**) Real-time qPCR measurement of DCT, TYR, and TYRP1B in 48 h post fertilization fish injected with either MC-GFP, MC-PPP6C or MC-PPP6C(R264C) (Student’s two-tailed t-test). All data panels in the figure are representative of at least three experiments. *p*-values are indicated as following: *p < 0.05, ****p ≤ 0.0001.

In order to investigate whether *PPP6C* regulates *MITF *in vivo we utilized genetic approaches in zebrafish. Using CRISPR, we performed targeted disruption of the PPP6C coding sequence in wild-type embryos. Targeted disruption of *ppp6c* led to embryonic lethality at 24 h post fertilization (Supplemental Fig. 1). This is consistent with prior research in mice showing the requirement of PPP6C for post implantation embryogenesis^8^. In order to study *PPP6C* specifically in the melanocyte compartment, expression via the MiniCoopR vector was used. The tol2 transposon-based MiniCoopR vector was used to express the human orthologue of PPP6C as well as the zebrafish orthologue of MITF (*mitfa*) open reading frame under control of the *mitfa* promoter (as described in Iyengar et al.^23^). The resulting vector was then injected into 1 cell stage embryos containing a mutation in *mitfa*^24^. Therefore, only fish in which the vector was successfully incorporated underwent melanocyte differentiation, allowing us to examine the effects of PPP6C expression in melanocytes. Vectors encoding PPP6C, PPP6C(R264C) and GFP (control) were created (referred to as MC-PPP6C etc.). Expression of PPP6C reduced the number of melanocytes on 5 day old fish as compared to GFP controls (Fig. 2A,B). Expression of PPP6C(R264C) further reduced the number of melanocytes. These fish were assayed for *mitfa* and target gene expression. While neural crest markers were unaffected, *mitfa* and *mitfa* target gene expression was reduced in fish expressing PPP6C and further reduced in fish expressing PPP6C(R264C) (Fig. 2C,D). These results indicate that in vivo expression of PPP6C affects melanocyte differentiation and results in a phenotype of fewer melanocytes.

**Figure 3:**
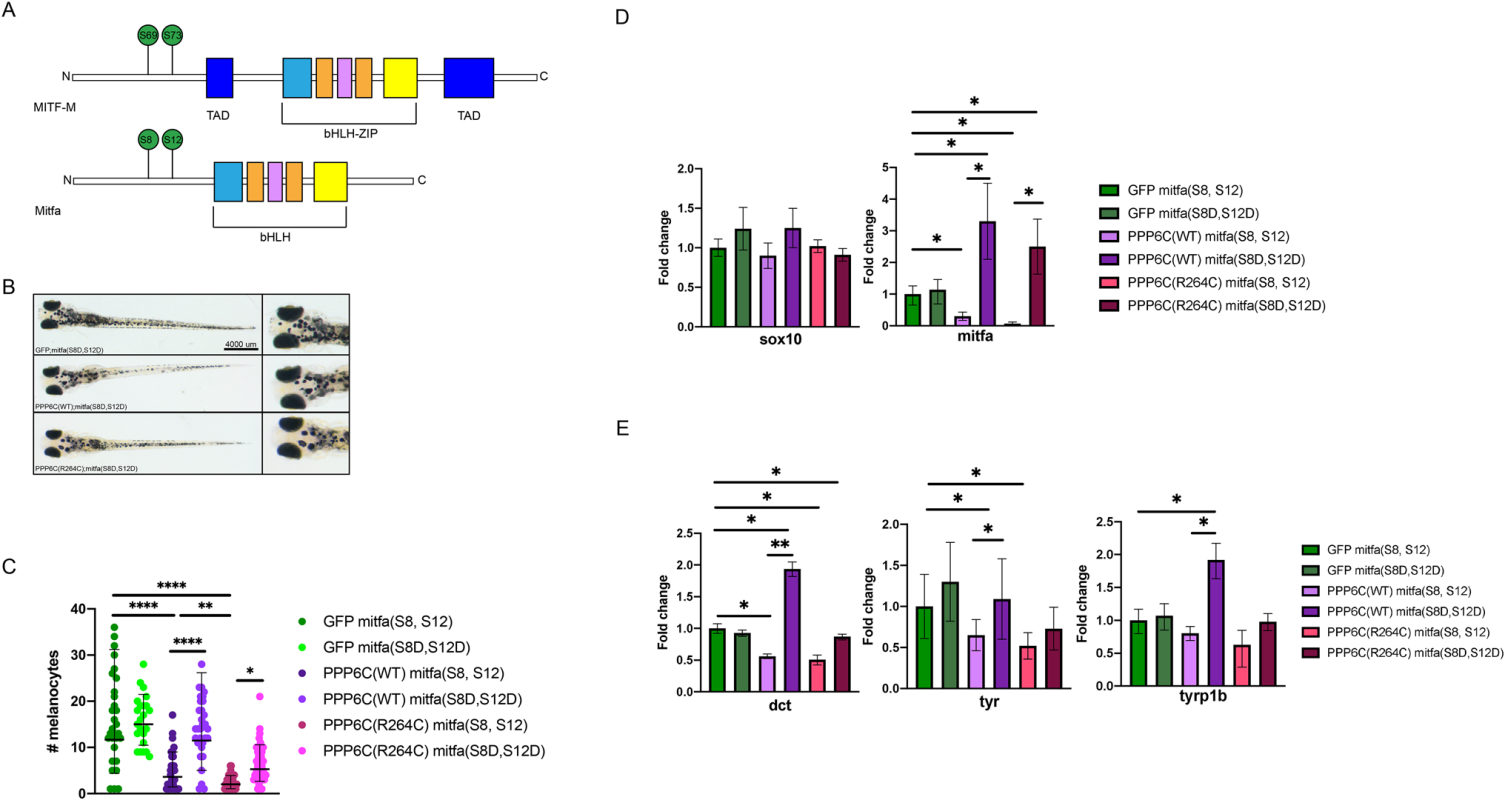
MITF mutation prevents PPP6C-mediated melanocyte differentiation disruption*.* (**A**) Schematic of the human MITF-M and zebrafish Mitfa proteins. (**B**) Representative images of 5 day old fish on the roy;nacre background injected with either MC-GFP;mitfa(S8D,S12D), MC-PPP6C;mitfa(S8D,S12D), or MC-PPP6C(R264C**)**;mitfa(S8D,S12D). (**C**) Representative counts of melanocytes of 5 day old fish on the roy;nacre background injected with either MC-GFP, MC-PPP6C, or MC-PPP6C(R264C) and with either MITF(S8,S12) or the MITF(S8D, S12D) mutations (Student’s two-tailed t-test). (**D**) Real-time qPCR measurement of SOX10 and MITF in 48 h post fertilization fish injected with either MC-GFP, MC-PPP6C or MC-PPP6C(R264C) and with either MITF(S8,S12) or the MITF(S8D, S12D) mutations (Student’s two-tailed t-test). (**E**) Real-time qPCR measurement of DCT, TYR, and TYRP1B in 48 h post fertilization fish injected with either MC-GFP, MC-PPP6C or MC-PPP6C(R264C) with and with either MITF(S8,S12) or the MITF(S8D, S12D) mutations. All data panels in the figure are representative of at least three experiments. *p*-values are indicated as following: *p < 0.05, **p ≤ 0.01, ****p ≤ 0.0001.

Prior research has shown that regulation of the phosphorylation status of MITF is important for its expression and activity, as different transcriptional programs are upregulated based on MITF levels. Phosphorylated MITF, regulated in part by KIT-mediated phosphorylation, is active, increases its own expression and upregulates a transcriptional program of melanocyte differentiation^16,25^. With this in mind, we hypothesized that there was an interaction between PPP6C and Mitfa, and used genetic approaches in zebrafish to test this hypothesis. Prior loss of function research has shown the serine residues S69 and S73 on MITF play a role in melanocyte differentiation^14,25,26^. These two residues are conserved in the zebrafish orthologue as S8 and S12 (Fig. 3A). We performed site-directed mutagenesis on the *mitfa* open reading frame to selectively mutate S8 and S12 to phosphomimetic aspartic acid residues (hereafter referred to as “*mitfa*(S8D,S12D)”). Injection of vectors containing *mitfa*(S8D,S12D) mutations resulted in a rescue of melanocyte number in fish expressing PPP6C or PPP6C(R264C) (Fig. 3B,C). At the molecular level, introduction of a phosphomimetic S8D,S12D *mitfa* also rescues *mitfa* and *mitfa* target gene expression (Fig. 3D,E). Together, these experiments indicate that PPP6C impacts Mitf phosphorylation during differentiation and the R264C mutation has an impact on melanocyte differentiation^27^.

### MITF promoter activity is reduced under expression of PPP6C(WT) and PPP6C(R264C)

**Figure 4:**
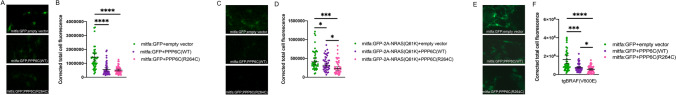
MITF promoter activity is reduced under expression of PPP6C(WT) and PPP6C(R264C) on multiple oncogenic backgrounds. (**A**, **C**, **E**) Representative confocal imaging of fluorescent pigment less melanocytes of tg*BRAF(V600E); nacre;p53(M214K);albino, roy;nacre,* or *roy;nacre;p53(M214K)* fish injected with a GFP reporter under control of the *mitfa* promoter. (**B**, **D**, **F**) Representative quantification of fluorescence for each genotype. Fish were imaged under a confocal microscope and total fluorescence was quantified for 10 melanocytes on N = 5 fish of each genotype. All data panels in the figure are representative of at least three experiments (Student’s two-tailed t-test). *p*-values are indicated as following: *p < 0.05, ***p ≤ 0.001, ****p ≤ 0.0001.

In order to directly study the effect of PPP6C on mitfa gene expression we developed a reporter gene assay in transgenic zebrafish embryos. MITF is able to transactivate its own promoter and participates in a positive feedback loop to maintain expression^28,29^. To measure *mitfa* promoter activity, we utilized the MiniCoopR encoding GFP. MiniCoopR vectors containing GFP and either PPP6C(WT) or PPP6C(R264C) were injected into fish containing albino and *mitfa* mutations, resulting in fluorescent and pigment less melanocytes, (Fig. 4). Melanocytes were imaged and fluorescent intensity was quantified to measure *mitfa* promoter activity. The effects of PPP6C on *mitfa* promoter activity were measured first on a non-oncogenic background. As compared to GFP controls, embryos containing either PPP6C(WT) or PPP6C(R264C) showed reduced GFP signal, consistent with reduced *mitfa* promoter activity (Fig. 4A). Next, we sought to determine if this reduced activity was consistent on both the BRAF and NRAS oncogenic backgrounds. Regardless of oncogenic background, melanocytes expressing PPP6C(WT) had reduced GFP expression and *mitfa* promoter activity (Fig. 4B,C). Additionally, embryos containing PPP6C(R264C) showed a further reduction in GFP expression and *mitfa* promoter activity. This data demonstrates that PPP6C has an effect on *mitfa* promoter activity in vivo, consistent with a negative effect on *mitfa* transcriptional activity. Additionally, the melanoma-specific oncogenic mutant PPP6C(R264C) led to a further reduction in expression of *mitfa*:GFP in vivo, suggesting that R264C is a gain of function mutation.

### The recurrent R264C mutation confers a gain of function proliferation phenotype in melanoma

**Figure 5:**
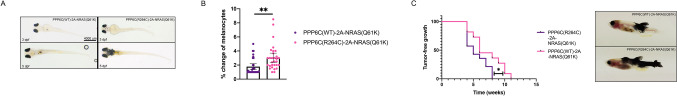
The recurrent *PPP6C*(R264C) mutation confers a gain of function proliferation phenotype in melanoma. (**A**) Representative images of fish injected with either PPP6C(WT)-2A-NRAS(Q61K) or PPP6C(R264C)-2A-NRAS(Q61K) at 3- and 5-days post fertilization. (**B**) Quantification of proliferation as measured by the change in number of melanocytes from 72 to 120 h post fertilization, N = 15 fish (Student’s two-tailed t-test). (**C**) Representative melanoma-free survival curve comparing tumors expressing PPP6C(R264C) to PPP6C(WT). One of two independent experiments is shown (log-rank (mantel-cox) test). *p*-values are indicated as following: *p < 0.05, **p ≤ 0.01.

We sought to determine the impact the R264C mutation has on cellular proliferation and tumor onset in a melanoma model, since low MITF expression has been shown to be oncogenic in a subset of melanomas^17,18^ and promotes tumor initiation in cultured melanoma cell lines^30^. Fish were injected with a vector co-expressing NRAS(Q61K) and either PPP6C(WT) or PPP6C(R264C). At 3 days and 5 days post fertilization, the number of melanocytes on the head of the fish were counted (Fig. 5A). The percentage change in number of melanocytes between days was calculated. PPP6C(R264C) led to a significant increase in melanocyte proliferation as compared to expression of PPP6C(WT) (Fig. 5B). Zebrafish expressing PPP6C(R264C) and NRAS(Q61K) developed tumors significantly faster than those injected with wildtype PPP6C and NRAS(Q61K) (Fig. 5C). Together, this data points to the role of PPP6C as a modulator of *mitfa* expression and suggests that expression of PPP6C(R264C) enhances melanoma initiation on an NRAS(Q61K) background.

### PPP6C expression affects MITF in melanoma

**Figure 6:**
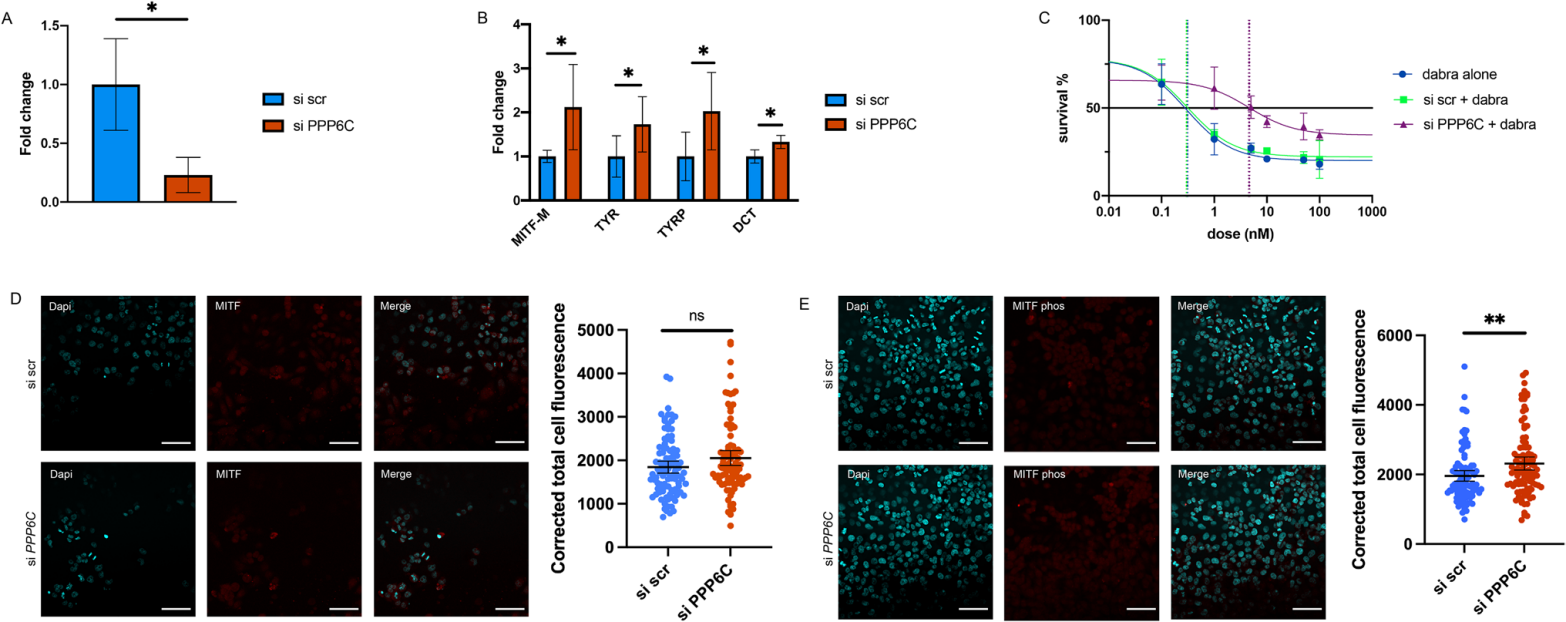
PPP6C expression affects MITF expression and drug resistance in melanoma. (**A**) Real-time PCR measurement of PPP6C in cells 72 h after transfection with either siPPP6C or siNon-targeting (Student’s two-tailed t-test). (**B**) Real-time PCR measurement of MITF and three target genes in cells with and without PPP6C knockdown 72 h after transfection (Student’s two-tailed t-test). (**C**) Drug dose response curve for A375 cells treated with dabrafenib and knockdown of PPP6C 72 h after transfection and treatment. Dotted lines display EC 50 for each condition. (**D**) Cellular expression of total MITF in A375 cells. Representative confocal images of fluorescence 72 h after transfection with either siPPP6C or siNon-targeting and staining with a MITF antibody, with quantification of fluorescence for each condition. One of two independent experiments is shown (Student’s two-tailed t-test). (**E**) Cellular expression of phospho-MITF in A375 cells. Representative confocal images of fluorescence 72 h after transfection with either siPPP6C or siNon-targeting and staining with a phospho-specific MITF antibody, with quantification of fluorescence for each condition. One of two independent experiments is shown (Student’s two-tailed t-test). *p*-values are indicated as following: *p < 0.05, **p ≤ 0.01. NS: non-significant *p*-value.

After observing that *mitfa* transcriptional activity is negatively affected by PPP6C expression, we sought to identify the changes that might occur when PPP6C expression is reduced in an MITF-low melanoma. We hypothesized that genetic inactivation of PPP6C would lead to higher levels of MITF and promote a more differentiated cellular state. In order to test this hypothesis, we employed the A375 melanoma cell line, which expresses very low levels of MITF^31^. Cells were transfected with an siRNA against PPP6C to reduce gene expression (Fig. 6A). MITF and MITF target gene expression was assayed. PPP6C knockdown increased MITF and MITF target gene expression significantly (Fig. 6B). Additionally, we measured the effect PPP6C expression has on drug sensitivity. A375 cells were treated with dabrafenib, a small molecule inhibitor of BRAF(V600E)^32^ and knockdown of PPP6C was performed (Fig. 6C). Cells treated only with dabrafenib or with a non-targeting siRNA displayed a robust response to treatment (EC50 = 0.3 nM). By comparison, cells treated with PPP6C siRNA showed a 15-fold decrease in sensitivity (EC50 = 4.6 nM). This data indicates PPP6C is able to modulate expression levels of *MITF* and induce drug resistance in a BRAF(V600E) mutant human melanoma cell line. Finally, we examined MITF expression and phosphorylation after PPP6C knockdown. We find no significant change in MITF protein expression, as determined by confocal microscopy after immunofluorescent staining after PPP6C knockdown (Fig. 6D). We do find an increase in phospho-MITF as measured by confocal microscopy after immunofluorescent staining of cells treated with PPP6C siRNA (Fig. 6E).

## Discussion

Our data reveals a novel role for PPP6C in regulating the activity of MITF in melanocytes and melanoma. In in vitro knockout and knockdown experiments, MITF and MITF target gene expression increased when PPP6C expression decreased, while proliferation decreased (Figs. 1C, 6B). In an in vivo zebrafish model this relationship was preserved, where PPP6C expression reduced *mitfa* and *mitfa* target gene expression significantly (Figs. 2C,D, 3D,E). Additionally, our data suggest that *PPP6C*(R264C) is a gain of function mutation in genetic assays investigating MITF function using zebrafish.

Phosphatases continue to emerge as important and druggable targets across multiple human cancers. SHP2, a tyrosine phosphatase involved in MAP kinase signaling acquires gain of function mutations such as D61Y that enhance its activity. These gain of function mutations are found in a variety of cancer types such as lung cancer, acute myeloid leukemia, and colon cancer^33^. Recently, SHP2 inhibitors such as SHP099 have shown promising efficacy in preclinical experiments and have entered clinical trials^34^. The recurrent R264C mutation in PPP6C may be another critical drug target as it is uniquely found in human melanoma.

Treatment of patients with BRAF(V600E) melanoma with BRAF and MEK inhibitors such as dabrafenib and trametinib have proven to prolong patient survival^2^. However, about half of patients do not respond to inhibition and show progression of disease. It has been shown that in some melanomas, reducing MITF expression can sensitize cells to chemotherapeutics^15^, consistent with the rheostat model of modulating MITF expression in melanoma. Our results show that knockdown of PPP6C and the associated increase in MITF leads to decreased sensitivity to BRAF(V600E) inhibition (Fig. 6C).

Regulation of MITF activity and phosphorylation are important melanoma initiation and progression. Higher levels of MITF expression are associated with cell cycle arrest and pigment-producing differentiation. As MITF levels decrease, programs associated with proliferation, survival, and invasion are upregulated; however, some baseline level of MITF expression is required to prevent senescence^35,36^. A growing body of evidence shows that in vivo, tumors switch between multiple MITF^high^ and MITF^low^-associated phenotypes in response to changes in the environment including BRAF inhibition, hypoxia or inflammation^37–39^.

Based on the data presented we suggest that PPP6C plays a role in disease modulation and phenotypic heterogeneity through its regulation of MITF. Expression of the gain of function mutation R264C cooperated with BRAF and NRAS oncogenes to reduce *mitfa* expression and led to an increase in melanoma proliferation in a zebrafish model system*.* This is consistent with prior studies that have shown that MITF haploinsufficiency leads to cell cycle stimulation. Mice heterozygous for MITF produce fewer melanoblasts, but these cells display greater proliferation during migration^40^. Additionally, we demonstrate that genetic inactivation of PPP6C affects sensitivity to BRAF inhibition, suggesting that PPP6C levels may be predictive of clinical response to BRAF inhibitors. Finally, we find that genetic inactivation of PPP6C leads to a modest increase in phosphorylated MITF. Further studies will be required to precisely identify the Ser/Thr residues targeted by PPP6C. Our data suggest that PPP6C plays a role in modulating MITF function as prior research has shown that MITF phosphorylation has an effect on both transcriptional activity and stability^26,41,42^. Together, these results define a unique role for PPP6C in melanocyte development and melanoma and place it upstream of regulating MITF expression and function.

## Methods

### TCGA data mining

All TCGA data sets for somatic mutations for cutaneous melanomas and other cancers were acquired from http://www.cbioportal.org.

### Preparation of transgenic vectors

All vectors were created using Gateway recombination (Life Technologies). A human PPP6C middle entry clone was made by PCR amplification as previously described^43^. pME-GFP, pME-PPP6C, pME-PPP6C-2A-NRAS(Q61K), and pME-GFP-2A-NRAS(Q61K) were used in LR reactions with pDEstTol2pA, p3E-polyA and p5E-mitf (containing a 2 kb of zebrafish mitfa promoter fragment) to generate pTol2mitfa:PPP6C-polyA, pTol2mitfa:PPP6C-2A-NRAS(Q61K)-polyA, pTol2mitfa:GFP-polyA, and pTol2mitfa:GFP-2A-NRAS(Q61K)-polyA. Throughout the manuscript MiniCoopR or MC vector refers to these pTol2 vectors. At each stage, vectors were Sanger sequenced (Genewiz) to verify correct orientation and sequence.

### Microinjection

For transgenic injections with a single vector, 250 pg vector and 50 pg transposase mRNA were injected into one-cell stage embryos. For transgenic injections with two vectors, 50 pg transposase mRNA, 250 pg MC-GFP and 500 pg MC-PPP6C(WT) or MC-PPP6C(R264C) were injected into one-cell stage embryos.

### Confocal imaging of fish

Embryos injected with MC-GFP were observed under a Zeiss SteREO Discovery.V8 scope at 46hpf for the presence of GFP fluorescence. Fluorescent embryos were imaged at 48hpf using a Zeiss LSM 800 Confocal microscope.

### GSEA

Melanoma cell line expression profiling files downloaded from Demeter2 Data v6 were analyzed by GSEA. GSEA was performed using the GSEA v4.0.3 software. PPP6C expression was used for enrichment of MITF target genes. All gene set files for this analysis were obtained from the Molecular Signatures Database v7.5.1. Enrichment map was used for visualization of the GSEA results. Enrichment score (ES) and False discovery rate (FDR) value were applied to sort MITF target genes enriched after gene set permutations were performed 1000 times for the analysis.

### RNA preparation and qPCR

Total RNA was extracted using Trizol reagent (Invitrogen) and the RNeasy Plus Mini Kit (QIAGEN) according to manufacturer’s instructions. 1 µg of total RNA was used for cDNA synthesis using the cDNA reverse transcription kit (Applied Biosystems) and incubated at 25 °C for 10 min followed by 120 min at 37 °C and 5 min at 85 °C. Real-time PCR was carried out using a LightCycler 480 II (Roche). Reactions were run in triplicate in two independent experiments. The variability in expression levels was normalized to the geometric mean of the internal control of housekeeping gene GAPDH. Primer sequences are listed in Supplemental table S1. Expression data were analyzed using the 2^−ΔΔCT^ method as described in Livak and Schimittgen, 2001.

### Site directed mutagenesis

Point mutations were introduced by PCR, using the QuikChange II XL Site-Directed Mutagenesis Kit (Agilent). Mutagenic primers were designed using Agilent’s QuikChange Primer Design and PAGE purified. The mutagenic primers used are shown in Supplemental table S1. PCRs for amino acid mutations were run for 18 cycles of 50 s at 95 °C followed by 50 s at 60 °C and 1 min/kb of plasmid length at 68 °C. The resulting mutant plasmids were verified by sanger sequencing (Genewiz).

### Cell culture

A375 cells were purchased from ATCC and cultured according to the manufacturer's instructions. All cells were maintained with 1% penicillin/streptomycin (Gibco) and 10% FBS (Denville Scientific).

### siRNA transfection and drug application

Reagents for siRNA knockdown of PPP6C were purchased from Ambion (PPP6C: Silencer 43527, non-targeting control: Silencer 4390843). The siRNA oligonucleotides were transfected with Lipofectamine 3000 (Thermo Fisher) at a final concentration of 100 pmol and incubated for 72 h.

### Immunohistochemistry

Living cells in culture were directly fixed in 4% paraformaldehyde for 15 min, followed with 10 min permeabilization in 0.1% Triton X-100. To perform immunofluorescence, cells were blocked in 3% BSA for one hour followed by immuno-staining with primary antibodies (pan MITF Cell Signaling D5G7V; phosho-MITF S73/180 ThermoFisher Scientific PA5104707) at 4 °C overnight 1:500 and secondary antibody (Invitrogen Alexa Fluor 647 goat anti-rabbit) at RT 1:500 for 1 h in 1% BSA. Nuclei were counterstained by DAPI (Thermo Fisher Scientific 62248). The figures were processed using Adobe Illustrator CC2017.

### Confocal imaging of cells

Cell after transfection with siRNAs and fixation were observed under a Zeiss SteREO Discovery.V8 scope. 4 fields with at least 25 cells per condition were examined for fluorescent intensity, for a total of at least 100 cells. Each image was 638.9 μm^2^. The frame size captured was 1024 pixels by 1024 pixels, with 8 bits per pixel. A 405 and 640 laser were used to capture the DAPI channel and the 647 channel for each field respectively. The DAPI channel had a pinhole of 45 μm and was set to 8.0% laser intensity. The master gain was set to 700 V, while the digital offset was 0 and the digital gain was 1.0. The 647 channel had a pinhole of 41 μm and was set to 4% laser intensity. The master gain was set to 740 V, while the digital offset was 0 and the digital gain was 1.0.

### Cell Titer Glo

Cells were plated at a concentration of 5000 cells onto 96 well plates and incubated at 1% FBS (Denville Scientific). Dabrafenib was purchased from Selleckchem. Cells were treated with dabrafenib at concentrations of 0.1–100 nM. At 48 h fresh drug was applied to cells. After 72 h cells were brought up to room temperature and 100 µL of CellTiter-Glo (Promega) reagent was added directly to each well. Plates were incubated on a shaker for 5 min and luminescence was measured on a Synergy 4 reader (Biotek). Luminescence readings were normalized to and shown as a relative percentage of DMSO control readings.

### Zebrafish husbandry

Zebrafish were bred and raised in accordance with established guidelines^44^. All experiments were carried approved by a protocol overseen by the Institutional Animal Care and Use Committee (IACUC) of Weill Cornell Medical College facilities. Additionally, all studies were in compliance with the ARRIVE (Animal Research: Reporting of in Vivo Experiments) guidelines.

### gRNA selection and preparation

Gene-specific guide RNAs were chosen using the GuideScan prediction tool^45^. The selected gRNA sequence was predicted to have no off targets with up to one base pair mismatch. crRNAs were synthesized by IDT as Alt-R CRISPR-Cas9 crRNAs. A bipartite synthetic gRNA was heteroduplexed using crRNAs and a tracrRNA according to IDT recommendations. Individual gRNAs were evaluated to ensure efficient induction of indels by microinjection (see description above) and by clonal analysis from a pool of injected embryos. The gRNA used induced indels in a minimum of 80% of clones analyzed from a pool of 10 embryos.

### Clonal analysis of gene editing

Genomic DNA was isolated from a pool of 20 embryos (24 h post-fertilization, or hpf) with DirectPCR Lysis Reagent (Viagen) and Proteinase K at 20 μg/ml (Qiagen). Samples were incubated at 55 °C for 60 min then 85 °C for 45 min. Genomic DNA was PCR amplified using primers outlined in Supplementary Table S1. 8 μl PCR product was mixed with 1.6 μl NEB Buffer 2 and 6.4 μl of water and was hybridized by incubation at 95 °C for 5 min then cooled from 95–85 °C at − 2 °C/s and 85–25 °C at − 0.1 °C/s. The hybridized DNA digested for 1 h at 37 °C with 2 U of T7EI endonuclease (New England BioLabs (NEB). Digested product was visualized on a 2% agarose gel.

To perform clonal analysis, the PCR product was cloned into pCRII-TOPO (Invitrogen) and subjected to Sanger sequencing (Genewiz). Sequences were compared to the danio rerio reference genome and uninjected controls to identify indels with the MacVector software (Version 16.0).

## Supplementary Information

Supplementary Information.

## Data Availability

The datasets generated during and/or analysed during the current study are available from the corresponding author on reasonable request.
