# Supplementary material for: PPP6C, a serine-threonine phosphatase, regulates melanocyte differentiation and contributes to melanoma tumorigenesis through modulation of MITF activity
Source: Sci Rep. 2022 Apr 2;12:5573. doi: 10.1038/s41598-022-08936-0 (PMC8976846; doi:10.1038/s41598-022-08936-0)
Supplement: Supplementary file 1 — Supplementary Information. [file 41598_2022_8936_MOESM1_ESM.doc]

**
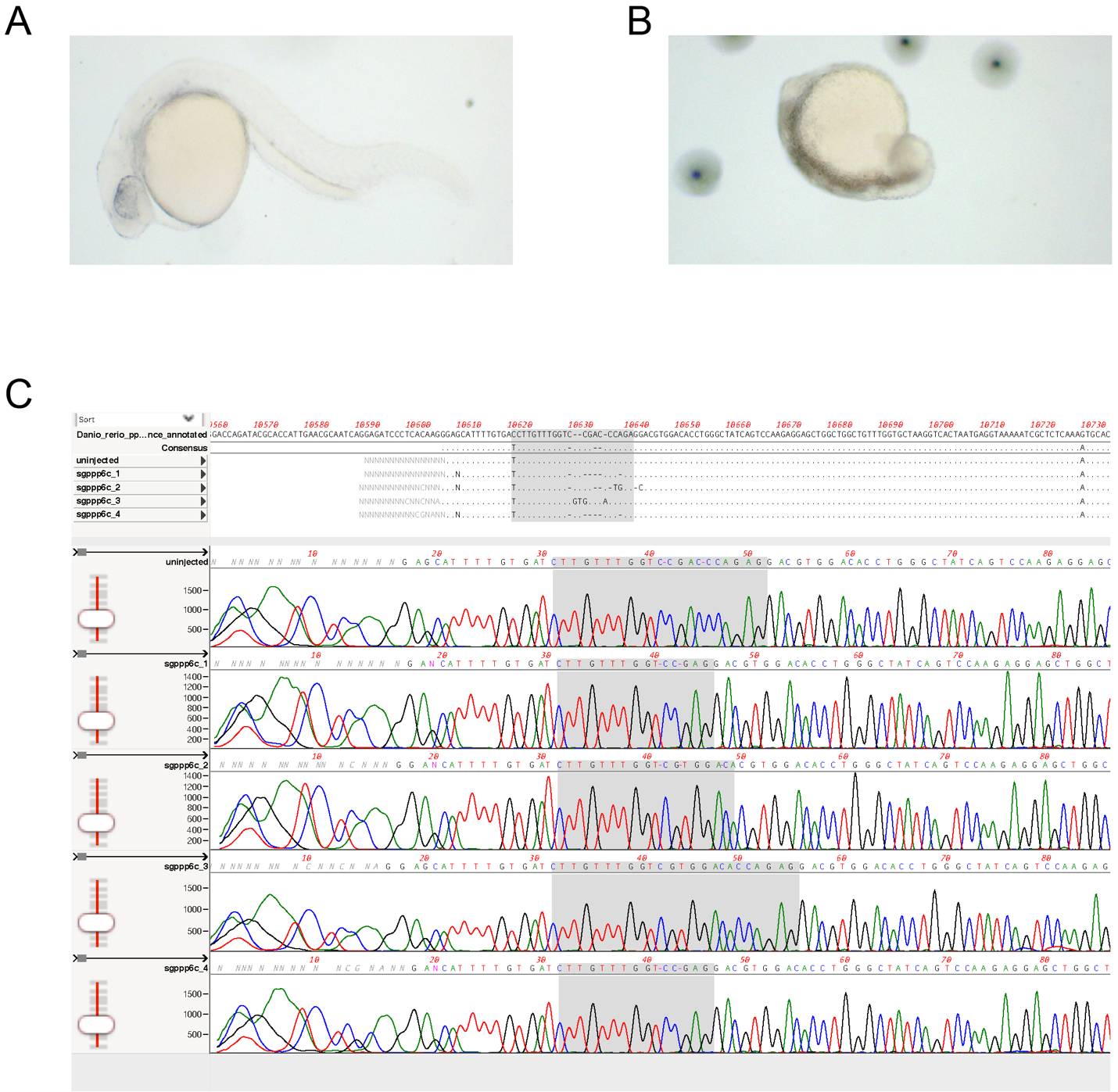
**

**Fig. S1. PPP6C Knockout is embryonically lethal in zebrafish**. **A-B)** Representative imaging of AB(WT) fish injected with 1000pg of guide against nontargeting genomic region (A) or *ppp6c* (B) 24 hours post fertilization. **C)** Clonal analysis of pool of 20 embryos shows editing at guide site of *ppp6c* locus (grey highlighted region).

Supplementary Table S1: List of Primers

**Oligo Name**

gapdh (F)

gapdh (R)

sox10 (F)

sox10 (R)

mitfa (F)

mitfa (R)

tyr (F)

tyr (R)

tyrp1b (F)

tyrp1b (R)

dct (F)

dct (R)

GAPDH (F)

GAPDH (R)

SOX10 (F)

SOX10 (R)

MITF (F)

MITF (R)

TYR (F)

TYR (R)

TYRP1 (F)

TYRP1 (R)

DCT (F)

DCT (R)

PPP6C (F)

PPP6C (R)

mitfa(S8D,S12D) mutagenic primer (F)

mitfa(S8D,S12D) mutagenic primer (R)

ppp6c sgRNA

**Sequence**

CCCAATGTCTCTGTTGTGGA

AACCTGGTGCTCCGTGTATC

TAACACTGCCGCACTACAGC

TGGAGTGGGCGTAGTAGGAT

GGTTCATGGATGCAGGACTT

GGGCAGGAGTTACTGATGGA

ATGAACGGCTCCATGTCTTC

CTGCTCAAAGATGCTGTCAATAAA

CTTCGACACACCACCGTATT

CTCACTCACATGGCTGTATTCT

AACCACCGTTCATCCTGATTAT

CCCGTCAGTTTCTGTAGTTCTC

GCAAATTCCATGGCACCGTC

TCGCCCCACTTGATTTTGG

CTTTCTTGTGCTGCATACGG

AGCTCAGCAAGACGCTGG

GGAGCTCACAGCGTGTATTT AGCGCATGTCTGGATCATTT

CTTACTCAGCCCAGCATCAT

GGGCGTTCCATTGCATAAAG

TCCACTCTAATAAGCCCAAACTC

CATCCTCGGTGCTGTTACAA

CCTGTCTCTCCAGAAGTTTGAC

CCAGAGTCCCATCTGCTTTATC

CAGCCAGTATCAACACCAGTAA

ACCTGACCTCCAGTTCTGA

﻿AGGGCCATAGGGCTGTTGGGAGCGCTGGCTCCGGGTCC

﻿GGACCCGGAGCCAGCGCTCCCAACAGCCCTATGGCCCT

﻿

GCCCTTCCTTTCTCCAGGAC

**Supplementary Table S1. List of primers.** Oligos used for qPCR, site directed mutagenesis, and CRISPR-Cas9-based targeting listed respectively. Zebrafish-specific primers are written in lowercase, and human-specific primers and written in uppercase.
